# Predicting the Impact of Long-Term Temperature Changes on the Epidemiology and Control of Schistosomiasis: A Mechanistic Model

**DOI:** 10.1371/journal.pone.0001438

**Published:** 2008-01-16

**Authors:** Tara D. Mangal, Steve Paterson, Andrew Fenton

**Affiliations:** School of Biological Sciences, University of Liverpool, Liverpool, United Kingdom; University of Liverpool, United Kingdom

## Abstract

**Background:**

Many parasites of medical and veterinary importance are transmitted by cold-blooded intermediate hosts or vectors, the abundance of which will vary with ambient temperatures, potentially altering disease prevalence. In particular, if global climate change will increase mean ambient temperature in a region endemic with a human pathogen then it is possible that the incidence of disease will similarly increase. Here we examine this possibility by using a mathematical model to explore the effects of increasing long-term mean ambient temperature on the prevalence and abundance of the parasite *Schistosoma mansoni*, the causative agent of schistosomiasis in humans.

**Principal Findings:**

The model showed that the impact of temperature on disease prevalence and abundance is not straightforward; the mean infection burden in humans increases up to 30°C, but then crashes at 35°C, primarily due to increased mortalities of the snail intermediate host. In addition, increased temperatures changed the dynamics of disease from stable, endemic infection to unstable, epidemic cycles at 35°C. However, the prevalence of infection was largely unchanged by increasing temperatures. Temperature increases also affected the response of the model to changes in each parameter, indicating certain control strategies may become less effective with local temperature changes. At lower temperatures, the most effective single control strategy is to target the adult parasites through chemotherapy. However, as temperatures increase, targeting the snail intermediate hosts, for example through molluscicide use, becomes more effective.

**Conclusions:**

These results show that *S. mansoni* will not respond to increased temperatures in a linear fashion, and the optimal control strategy is likely to change as temperatures change. It is only through a mechanistic approach, incorporating the combined effects of temperature on all stages of the life-cycle, that we can begin to predict the consequences of climate change on the incidence and severity of such diseases.

## Introduction

Global climatic changes alter the equilibrium of many ecosystems and the distribution of species they support [1], [2]. In particular, the potential effects of climate change on the distribution and severity of human diseases are of major interest [3]–[7]. These changes can arise through both direct effects of climate change, i.e. alterations in the geographic areas able to support disease vectors, and indirect effects, such as changes in human migration patterns affecting disease distribution. Furthermore, the impacts of climate change may occur over several time scales, ranging from increasing the amplitude and stochasticity of diurnal or seasonal fluctuations in temperature and precipitation, particularly in temperate regions, to more stable increases in mean ambient temperatures over longer periods, particularly in tropical regions where many of the more concerning human diseases are endemic. The regions most vulnerable to the disease-related impacts of climate change are the temperate latitudes and the countries in the Indian and Pacific Oceans and sub-Saharan Africa, which will be disproportionately affected by extremes in temperature, and where public health programmes may be unable to cope with the changes in disease transmission [8].

Of particular concern is the impact climate change will have on the prevalence of many vector borne infectious diseases, including malaria, schistosomiasis and dengue. The prevalence and abundance of these vector-borne diseases are particularly sensitive to changes in mean ambient temperature since their transmission relies principally on the survival and reproduction of their invertebrate vector or intermediate host, and the parasite's incubation and survival rates therein. Since these vectors and intermediate hosts are incapable of thermoregulation, and their reproduction and survival rates are strongly influenced by temperature, small changes in temperature could greatly alter their distribution and abundance, resulting in a shift in disease patterns. Predicting how the long-term distribution and prevalence of such important human diseases will change in the face of global warming is a key challenge facing humans in the near future.

There are three main methods of examining the relationship between mean ambient temperature and infectious diseases. The first is to study current variations in climate and monitor the short-term effects on disease transmission. The second - a phenomenological approach - is to analyse past and current disease patterns and extrapolate these patterns into the future. The third method, which will form the focus of this paper, is to use mechanistic models to predict the changes in prevalence and the burden of infectious diseases in response to forecasted global warming scenarios. This mechanistic approach has the advantage that it allows greater confidence in extrapolating beyond current conditions into a range of possible future climate scenarios. Here, we adopt such an approach to predict the impact of long-term temperature changes on the prevalence and abundance of human schistosomiasis, caused by the trematode *Schistosoma mansoni*. Currently, 600 million people are at risk of infection by schistosome species and current research predicts that vector-borne diseases such as schistosomiasis will be particularly affected by changes in temperature [5], [9]. We specifically focus on long-term changes in mean ambient temperature, rather than short-term diurnal or seasonal temperature fluctuations, and we concentrate on the impact of increasing mean ambient temperatures on the snail-schistosome interaction within a region in which schistosomiasis is already endemic, rather than the spread of the disease into new areas. Ultimately, however, this approach can be extended to include geographical and ecological factors using a geographic information systems (GIS) approach to predict how the distribution of schistosomiasis will change in response to increased temperatures. Similar approaches have been developed for malaria, showing that increasing the average global temperature by 2–3°C, would increase the number of people at risk of infection worldwide by several hundred million [8].

**Figure 1:**
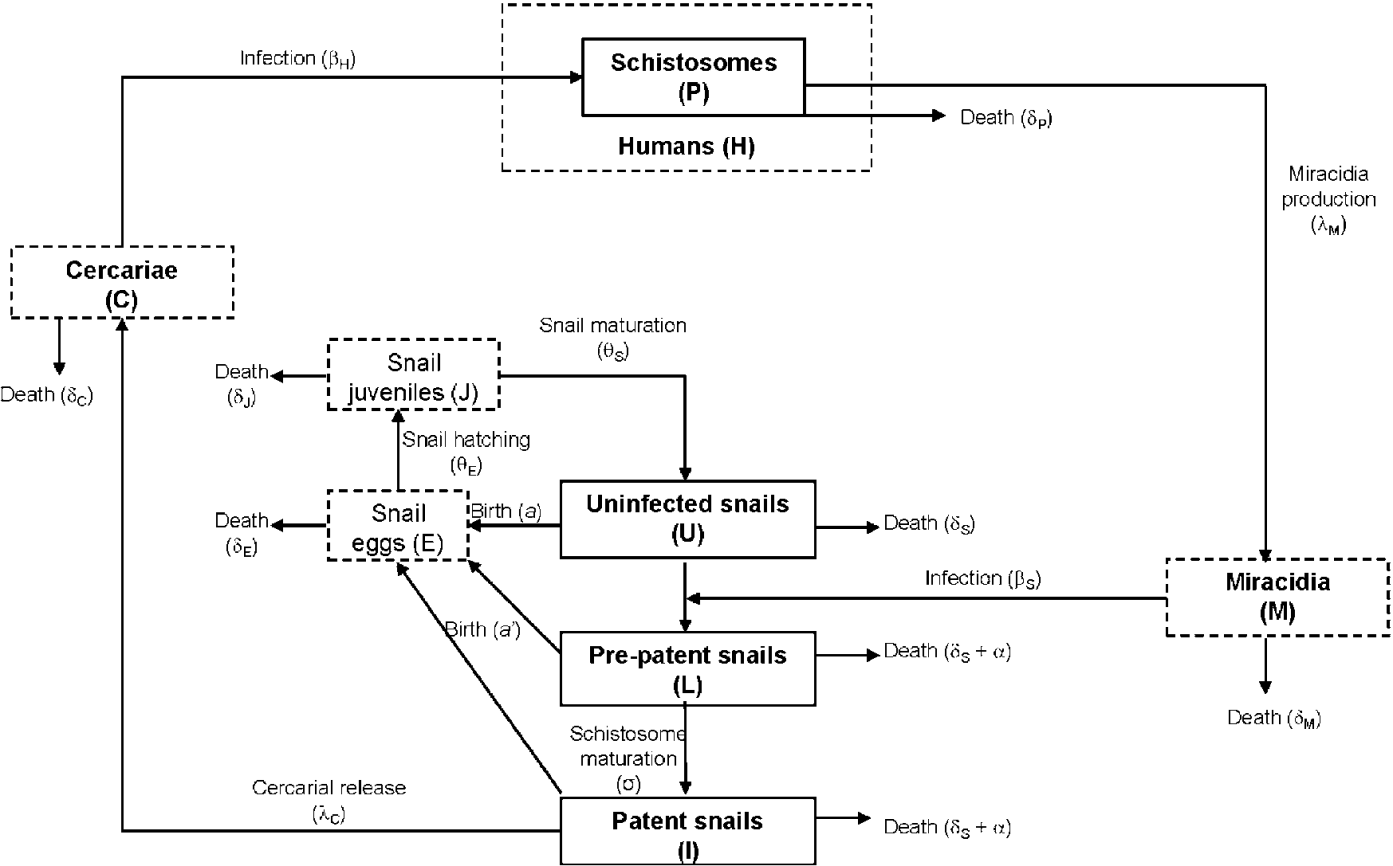
Schematic diagram of the schistosome disease model. The dashed boxes show stages of the life-cycle that are not modelled explicitly.

A number of mathematical models have been developed to understand the epidemiology, transmission dynamics and impact of control strategies on schistosomiasis [10]–[15]. The life-cycle of schistosomes is complex, involving two free-living stages and two host populations (Fig 1). Briefly, paired male and female adults in the (human) definitive hosts produce eggs which pass into the environment in the host's faeces. These eggs hatch into miracidia which seek out and infect the snail intermediate host where they undergo asexual reproduction. After a period of development, thousands of free-swimming infective cercariae are released, which actively seek out and penetrate a new human host, where they develop into adults. There are a number of stages of the schistosome and snail life cycles that will be highly dependent on ambient temperatures, affecting the distribution and prevalence of schistosomiasis and the likely response to global warming. Poulin [16] recently reviewed the effects of temperature on cercarial emergence and found that a 10°C temperature increase resulted in an average 8-fold increase in cercarial output. From this we may expect a large increase in the number of humans infected due to the large increase in cercarial production. However, Poulin focussed on only one stage of the parasite life-cycle (cercarial emergence) and ignored other key stages and potential sources of density dependent regulation within the parasite life-cycle. It is likely that different stages will respond in different ways to temperature increases, making it necessary to develop an explicit epidemiological model to predict how these factors combine to affect the overall impact of temperature changes on the host-parasite system. This paper addresses three important questions regarding the impact of temperature on schistosomiasis: What effect does temperature have on the prevalence of schistosomiasis in a population? How do long-term temperature increases affect the mean worm burden in the population? What are the implications of long-term temperature increases on the optimal control strategy of schistosomiasis in the field?

## Methods

Our schistosomiasis epidemiological model is modified from those developed by Woolhouse [12], [13] and Anderson and May [17] and is shown schematically in Fig. 1. Adult schistosomes within infected human hosts produce eggs which hatch and develop to free-swimming miracidia at a net rate *λ_M_*. These miracidia either die at rate *δ_M_* or infect uninfected snails (*U*) at rate *β_S_*. Due to the intense density dependence acting on schistosome development within snails, we follow previous models of schistosome epidemiology [12], [13] by ignoring the burden of infection within snails, and simply class snails as uninfected (*U*), latently (pre-patent) infected snails (*L*) and patent infected snails (*I*). Pre-patent infections develop to patency at rate σ, after which they release cercariae (*C*) at a constant rate *λ_C_*, which either die at rate *δ_C_* or infect human hosts (*H*) at rate *β_H_*. Successfully infecting schistosomes are assumed to mature immediately to adult parasites (*P*), which die at rate *δ_P_* and produce new eggs throughout their life to begin the life-cycle again. Based on empirical data from the literature (see below), we modify this basic model to incorporate more details on the snail life-cycle. Specifically, we consider the density of snail eggs (*E*) and the density of juvenile snail stages (*J*) in the environment. All adult snails may lay eggs, although infected snails may lay eggs at a different rate (*a*') from uninfected snails (which lay at rate *a*), but overall egg production of the population is limited by density dependent regulation by a carrying capacity, *K*. Snail eggs die at rate *δ_E_* and hatch at rate *θ_E_* and juvenile snails die at rate *δ_J_* and mature to adult, uninfected snails at rate *θ_S_*. All adult snails die at background rate *δ_S_* and infected adult snails also die due to parasite-induced mortality at additional rate *α*. All parameters are defined in Table 1.

**Table 1:** Estimates of each parameter at baseline temperatures of 20, 25, 30 and 35°C.

Parameter	Definition	20°C	25°C	30°C	35°C
λ_C_	Cercarial production rate	2476 d^−1^	4128 d^−1^	6103 d^−1^	8400 d^−1^
δ_C_	Cercarial mortality rate	1 d^−1^	1 d^−1^	1 d^−1^	1 d^−1^
β_H_	Cercarial infection rate	0.028 L^−1^ d^−1^	0.059 L^−1^ d^−1^	0.091 L^−1^ d^−1^	0.122 L^−1^ d^−1^
δ_P_	Adult schistosomes death rate	0.0309 d^−1^	0.02 d^−1^	0.01 d^−1^	0.008 d^−1^
λ_M_	Net miracidial production rate	500 d^−1^	500 d^−1^	500 d^−1^	500 d^−1^
δ_M_	Miracidia death rate	2 d^−1^	2.526 d^−1^	4.364 d^−1^	4.444 d^−1^
β_S_	Miracidia infection rate	1.27×10^−4^ L^−1^ d^−1^	9.1×10^−5^ L^−1^ d^−1^	1.4×10^−3^ L^−1^ d^−1^	1.2×10^−3^ L^−1^ d^−1^
σ	Within-snail schistosome maturation rate	0.0216 d^−1^	0.036 d^−1^	0.05 d^−1^	0.065 d^−1^
*a*_S_	Snail egg laying rate	0.663 d^−1^	0.849 d^−1^	0.057 d^−1^	0.010 d^−1^
θ_E_	Snail hatching rate	0.08 d^−1^	0.1 d^−1^	0.118 d^−1^	0.128 d^−1^
θ_S_	Snail maturation rate	0.02 d^−1^	0.029 d^−1^	0.012 d^−1^	0.0075 d^−1^
δ_E_	Snail egg mortality rate	0.001 d^−1^	0.001 d^−1^	0.001 d^−1^	0.001 d^−1^
δ_J_	Snail juvenile mortality rate	0.002 d^−1^	0.0038 d^−1^	0.0071 d^−1^	0.0207 d^−1^
δ_S_	Snail adult mortality rate	0.004 d^−1^	0.003 d^−1^	0.008 d^−1^	0.0182 d^−1^
α	Additional snail mortality due to infection	0.002 d^−1^	0.0145 d^−1^	0.0295 d^−1^	0.05 d^−1^
K	Snail carrying capacity	100 L^−1^	100 L^−1^	100 L^−1^	100 L^−1^

We simplify this detailed model by recognising that the dynamics of external stages of the schistosome life-cycle tend to be far quicker than those of the infecting stages, or their hosts [18]. We therefore assume the miracidia and cercariae are at ‘pseudo-equilibrium’ and do not model them explicitly. Similarly, we assume the dynamics of the egg and juvenile stages of the snail life-cycle are relatively fast and do not model them explicitly. Finally, we make the important assumption that the size of the definitive host population (humans) is constant, allowing us to consider the risk of infection to a static host population of a given size. This results in the final model comprising four state variables describing the adult size of the adult parasite population within humans (*P*), the density of uninfected snails (*U*), the density of latently infected snails (*L*) and the density of patently infected snails (*I*):
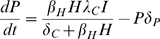
(1)

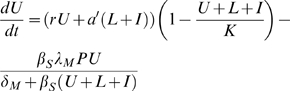
(2)

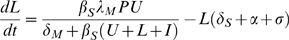
(3)

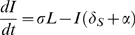
(4)


We parameterised this model at different temperatures using data from the literature which explored the effects of temperature on various life-history traits of both parasites and hosts (see online supplementary information). We then used these values to generate predictions of how the mean number of parasites per human host (*m = P/H*) and the prevalence of infection (*p*) change with temperature. For this latter measure we assume adult parasites are distributed among hosts in an aggregated manner, according to a negative binomial distribution with aggregation parameter *k*. Given the predicted mean burden of parasites per host, *m*, the predicted prevalence is given by:
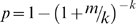
(5)


Clearly the data from the literature were not consistent for all life-history traits, and may have been collected for different species under different conditions, but we use them as a first approximation of parameter values. To explore how robust our predictions are to variations in parameter values we conducted a sensitivity analysis, where the value of each parameter in turn was increased up to 10 times from the baseline value and the relative impact on the predicted mean parasite burden and prevalence was calculated. This allows us to qualitatively determine the key parameters that need to be estimated accurately if we are to obtain reasonable predictions of the impact of temperature on schistosomiasis. Furthermore, this process reveals ‘leverage points’ in the epidemiology, highlighting traits that may be targeted by control measures to bring about the greatest reduction in disease. Once again, we emphasise that we take a broad brush approach, concentrating on the impact of changes in long-term mean ambient temperature on the abundance and control of schistosomiasis, rather than the impact of short-term diurnal or seasonal fluctuations in temperature. Hence, in what follows, the ambient temperature was assumed to remain constant throughout each simulation.

## Results

### The impact of ambient temperature on schistosome dynamics

**Figure 2:**
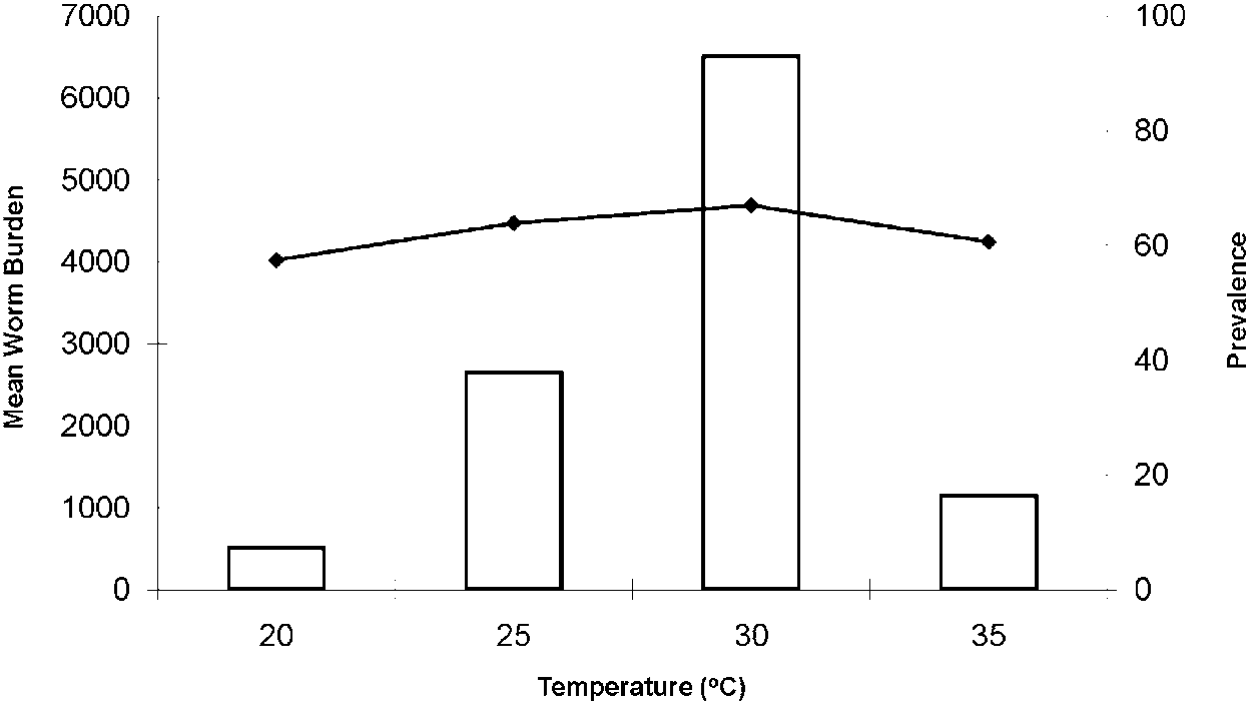
Variations in mean worm burden (bars) and prevalence (lines) of *Schistosoma mansoni* as a function of temperature.

The model predicts that mean worm burdens in humans are greatly affected by ambient temperature, rising to a peak at 30°C and then falling sharply at 35°C (Fig. 2). This decline in worm burdens at 35°C may be explained by the increasing mortality of both the intermediate hosts and the parasite at higher temperatures (see Appendix S1). However, the disease prevalence in humans remains almost constant over the temperature range of 20–35°C (Fig. 2), suggesting that although increases in long-term mean ambient temperature may lead to an increase in mean worm burdens and associated increases in morbidity and even mortality of infected hosts, the number of infected people is unlikely to change greatly.

**Figure 3:**
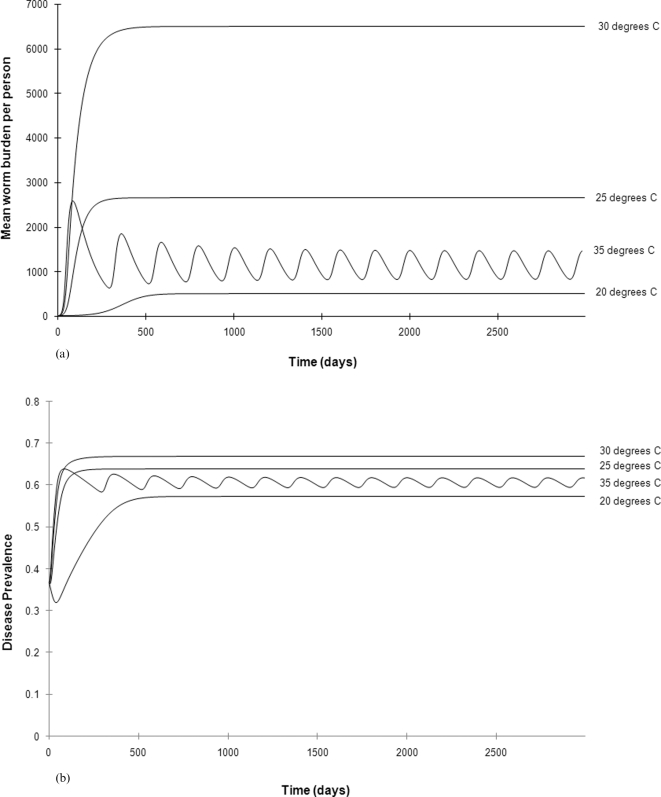
a. Dynamics of mean worm burden per individual over time at 20°C, 25°C, 30°C and 35°C. b. Dynamics of (b) prevalence of infection over time at 20°C, 25°C, 30°C and 35°C.

Interestingly, increased temperatures also lead to changes in disease dynamics (Figs. 3a and 3b). At 20°C, disease dynamics are stable, with mean burdens and prevalence remaining constant over time. However, at 35°C the dynamics switch from endemic to epidemic, with repeated oscillations in worm burdens and disease prevalence over time. A previous model of schistosome dynamics showed that density-dependent regulation of the intermediate host (snail) population could allow such cyclical dynamics [19] and our parameterised model shows that the interaction between such regulation and the increased snail birth rates at higher temperatures make such dynamics more likely.

**Figure 4:**
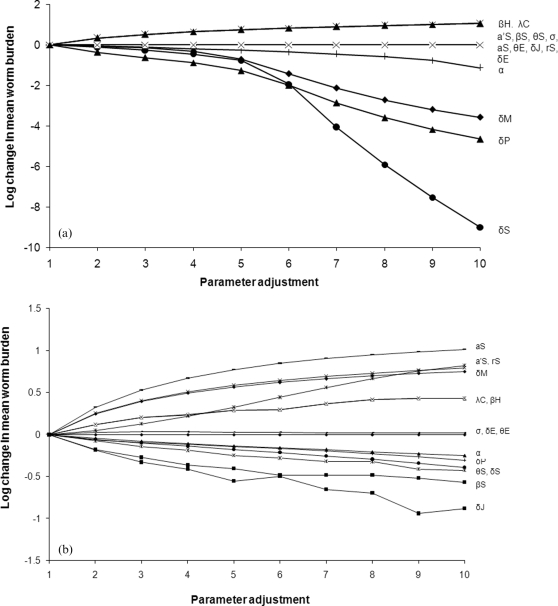
a. Results of the sensitivity analysis of the mean worm burden per individual at (a) 20°C. Each parameter was varied up to 10 times the value in the baseline model and the impact on the parasite population was assessed as the Log_10_ of the mean worm burden or prevalence following the adjustment divided by the value obtained using the baseline parameter values. b. Results of the sensitivity analysis of the mean worm burden per individual at 20°C and 35°C.

A sensitivity analysis was conducted where each parameter at each temperature was increased by up to a factor of 10 and the impact on mean worm burden per individual and prevalence measured (note that the results for these are qualitatively similar, so only the results for mean worm burden are presented here). By subjecting each parameter at each temperature to the same perturbation in turn this analysis allows us to rank the parameters in terms of their relative sensitivities in the model, and also provides insight into the impact of uncertainties in our estimation of the temperature-dependent relationships in the model. We also decreased each parameter by a factor of up to 10 and found that the ranking importance of each parameter remained the same as for the previous analysis and so for simplicity we do not present those results here. The most sensitive parameters at 20°C were the mortality rates of the adult snails (*δ_S_*), miracidia (*δ_M_*) and adult parasites (*δ_P_*) (Fig. 4a). Interestingly, however, the ranking of parameters varied with temperature; at 35°C the most sensitive parameters were the mortality rate of juvenile snails (*δ_J_*), the infection rate of snails (*β_S_*) and the birth rate of snails (*a_S_*) (Fig. 4b). All of these parameters involve snail life stages, highlighting the importance of the intermediate host in determining the dynamics and abundance of schistosomes, suggesting that control methods that target the snails will prove highly effective at increased ambient temperatures. Overall, these results show that the optimal control approach will vary depending on the ambient temperature and the degree of temperature change. For example, treating humans to increase adult parasite mortality may be an effective control strategy at 20°C, whereas it is likely to be a less effective control approach than increasing snail mortality at 35°C.

### The impact of ambient temperature on the optimal control strategy

The mathematical modelling of *S. mansoni* transmission can greatly aid in the development of control strategies both at the local and national level. We explored five separate control programmes; targeting the adults parasites using mass chemotherapy of the population (increasing *δ_P_*), targeting the snail intermediate hosts using molluscicides (increasing *δ_J_* and *δ_S_*), increasing sanitation (reducing *λ_M_*), and two combined approaches (chemotherapy combined with intermediate vector control), and a three-tiered approach combining chemotherapy and molluscicide use along with increased sanitation at four temperatures, 20, 25, 30 and 35°C. Control applications were assumed to be applied continuously, where the appropriate parameter in the model was altered by a factor of 2 (i.e., *δ_P_*_,_ *δ_J_* and *δ_S_* were doubled and *λ_M_* was halved). The model was run for 3000 days and the mean prevalence and mean parasite burdens in humans were recorded over this time.

**Figure 5:**
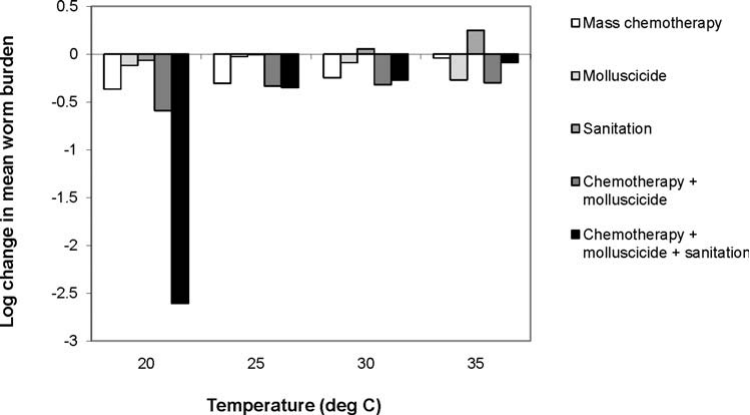
Impact of temperature on the efficacy of different control programmes: (1) targeting only the adult parasites by mass chemotherapy (achieved by doubling *δ_P_* from the baseline value); (2) targeting only the snail intermediate vectors through molluscicide application (by doubling *δ_J_* and *δ_S_*); (3) improved sanitation (by halving *λ_M_*); (4) combining intermediate vector control with mass chemotherapy and (5) combining intermediate vector control, mass chemotherapy and improved sanitation. The efficacy of control was measured as the Log_10_ of the mean parasite abundance during the control phase divided by the mean abundance in the absence of control.

As before, the responses to control of disease prevalence were qualitatively similar to those of mean worm burden, so we only present the latter results here. At 20°C, all control programmes showed a significant decrease in mean worm burden, which fall from an average of 504 worms per person in the absence of control to 217 for mass chemotherapy alone and to 1.2 for a programme combining chemotherapy, sanitation and intermediate vector control (equivalent to a 99% reduction in worm abundance, Fig. 5). However, the efficacy of each programme varied according to the ambient temperature; a combined chemotherapy and vector control programme became the optimal strategy at 25–35°C, whereas targeting adult parasites alone had a negligible effect at 35°C. Surprisingly, sanitation appeared to have a detrimental effect, almost doubling the burden of disease at 35°C. This arises due to the complex interaction between the strong density dependent constraints acting on cercarial production and the negative impact of schistosome infection on the snail population; reducing the input of miracidia into environment has little impact on the total number of cercariae released, since the same number are released per infected snail regardless of the number of infecting miracidia. However, reducing the number of miracidia also reduces the number of snails dying due to infection, allowing the snail population to build up to large numbers. This provides more susceptible snails for infection, resulting in an overall increase in the total number of cercariae being released into the environment and a subsequent increase in human infections. Overall, these results show a clear advantage to targeting both intermediate vectors and the human population over treating humans alone; indeed, it appears adopting a human-only treatment is very unlikely to be effective in reducing either the regional prevalence or mean worm burden of schistosomiasis at increased temperatures.

## Discussion

Predicting the impact of climate change on the epidemiology of infectious disease is a pressing challenge. However, extrapolating from current scenarios into the future is unlikely to be straightforward. Due to the differential impact of temperature on each of the life-history stages of a parasite there is unlikely to be a simple relationship between ambient temperature and disease prevalence [4]. Here we used a mechanistic epidemiological model, parameterised from the literature, to predict how increases in long-term mean ambient temperature will impact on the prevalence and abundance of *S. mansoni*, the primary causative agent of human schistosomiasis, within an endemic area. We showed that increasing ambient temperatures from 20°C to 30°C would result in a more than tenfold increase in the mean burden of infection. However, temperatures above 30°C are predicted to result in a decrease in the burden of disease, primarily through increased mortality rates of the snail intermediate hosts. Furthermore, our model showed that the prevalence of infection is unlikely to vary greatly regardless of the ambient temperature. Therefore, although the burden of infection will increase substantially within infected people which presumably results in increased morbidity and mortality rates, the number of people infected is unlikely to change. Nevertheless, the increased morbidity will have severe consequences, ranging from reduced birth rates, neurological complications and high economic costs through lost work days and the need for medical care.

The model quantifies human disease burden by predicting the mean number of worms harboured in an individual in the population. It would be interesting to validate the model's predictions by comparing existing worm burdens from regions with different ambient temperatures. However, this is not possible at present for two reasons. Firstly, patterns of worm burden from different regions will be influenced by a range of factors such as differences in human, snail and parasite genotypes, variations in microclimate and other environmental variables, and differences in human behaviour and exposure. Secondly, it is accepted that direct quantification of worm loads is impossible and so many studies use faecal egg counts to estimate the intensity of infection. While this is a very useful measure, the relationship between eggs per gram of faeces and worm load is continually disputed [20]. It is therefore extremely difficult to directly validate the model's predictions of worm load but rather we use the data as an indication of disease burden. The only available data containing actual measurements of worm pairs in individuals shows that counts of over 1000 may be rare [21], although this study includes only older age groups in a low transmission area and so generalising to other populations or regions is not feasible.

Current estimates state that a global temperature increase of 2–3°C over the next 100 years is likely [8]. However, by exploring a wider range of temperature values (20 to 35°C) we have shown that the impact of even a small rise in temperature on both prevalence and burden of infection will depend greatly on the initial, baseline temperature. In particular, an important result to emerge from our sensitivity analyses is that changes in mean ambient temperature will alter the relative sensitivities of the parameters in the model. Therefore the optimal disease control strategy will change as temperatures increase. In particular, at 20°C increasing adult parasite mortality rates, for example through chemotherapy, may have a large impact on the prevalence and abundance of disease, whereas at 35°C parameters relating to the snail stages become more important, suggesting that snail eradication programmes may be more successful. Indeed, the model showed that a combined approach integrating chemotherapy treatment with snail control had a larger impact on schistosome prevalence and abundance than chemotherapy alone. Macdonald [22] suggested that very high sanitation levels (i.e. reducing the number of eggs reaching water), has a negligible effect on mean worm load compared to the combined effects of treating infected people and keeping them out of infected water. This was because the water that the snails live in is typically saturated with miracidia and nearly all snails are infected. This is supported by our model which predicts that prevalences in the snails typically approach 100%, and so reducing human contact with the water is crucial for breaking the transmission cycle.

A number of control programmes, such as mass chemotherapy, molluscicide applications and improvements in sanitation and water supplies have been used in a combined effort to control schistosomiasis worldwide. These programmes have been successful in Brazil, the Middle East and parts of the Far East, but the disease has remained endemic in many regions of sub-Saharan Africa [9]. Many countries with endemic levels of schistosomiasis did not implement control programmes, believing the costs required for control would be disproportionately high compared with the health benefits. A number of control programmes were initiated in sub-Saharan Africa but local and national health authorities were unable to maintain the high costs involved. These programmes enjoyed short-term success but infection levels soon returned to pre-intervention states. In particular, Sturrock [23] noted that chemotherapy alone does not have a lasting effect on transmission, and suggested that re-infection rates largely depend on ecological factors affecting the snail population. Our model confirms this suggestion, particularly in regions with high ambient temperatures.

Clearly the design of any control programme needs to take into account a wide range of social, medical and environmental factors beyond the scope of our model (for example, the occurrence of adverse side effects to chemotherapy or the knock-on effects of mass molluscicide treatment on the wider ecological community). Furthermore, the model omits a number of biological complications, such as heterogeneous transmissions rates, the presence of alternative reservoir hosts and the build-up of acquired immunity [12], [13], [24]. One important simplification is that we restrict ourselves to considering the impact of long-term changes in mean ambient temperature on schistosome abundance and control. However, climate change is likely to have a number of impacts on the environment, including increased fluctuations in temperature over shorter time scales (e.g., diurnal or seasonal), and will also impact on the distribution and longevity of suitable water bodies for the snails. Such complications will modify the finer predictions of our model, but we believe our approach is appropriate for providing an initial insight into the broader consequences of climate change. Further studies, building on this existing framework and incorporating some of these factors would be invaluable. In particular, to improve the accuracy of the model, it is essential to conduct a series of experiments using one specific host-parasite combination over a range of temperatures. These could then be compared with field studies conducted in different regions with different ambient temperatures to validate the model. A fascinating next step would be to place this mechanistic model within a spatially explicit GIS framework with realistic migration patterns and predicted temperature regimes. This could further extend the work of Malone *et al* [25]–[27] who have used satellite imagery and geographic information system to develop area suitability maps and environmental risk models for schistosomiasis. It would then be possible to predict with greater confidence than current, extrapolation-based approaches how the spatial distribution of schistosomiasis will change under global warming. However, the current model provides an important mechanistic insight into how the complex interactions between the various life-history stages and ambient temperature will determine the impact of schistosomiasis and the success of future control programmes in the face of global climate change.

## Supporting Information

Appendix S1 Parameter estimation (0.10 MB (undefined))
