# Supplementary material for: Predicting the Impact of Long-Term Temperature Changes on the Epidemiology and Control of Schistosomiasis: A Mechanistic Model
Source: PLoS One. 2008 Jan 16;3(1):e1438. doi: 10.1371/journal.pone.0001438 (PMC2190776; doi:10.1371/journal.pone.0001438)
Supplement: Appendix S1 — (0.10 MB (undefined)) [file pone.0001438.s001.doc]

**Appendix S1: Parameter estimation**

We parameterise our model using all available data from the literature, where laboratory experiments were conducted at a range of temperatures (described below). We then extrapolated from these parameter estimates at the range of temperatures shown to obtain estimates of parameter values at our baseline temperatures of 20, 25, 30 and 35°C. Clearly, these studies were conducted under a range of conditions, sometimes using different snail or parasite species. It would therefore be invaluable to conduct comprehensive experiments using specific host-parasite species combinations at the full range of temperatures to obtain more accurate parameter estimates. However, we use the present data as a baseline with which to parameterise our model, fully acknowledging their limitations.

Estimating mortality rates

*1) Snail mortality rates (δ_J_ and δ_S_)*

El-Hassan (El Hassan, 1974) presented mortality rates for juvenile and adult snails at a range of temperatures over a two-week period. These can be converted into mortality rates per day using the following equation:

$\delta= -\frac{\ln(N_{t}/N_{0})}{t}$ Eq. A1

where *N_t_* is the number alive at time *t*. This resulted in the following estimates:

| Temperature (°C) | Juvenile mortality rate (*δ_J_*) | Adult mortality rate (*δ_S_*) |
| --- | --- | --- |
| 10 | 0.0080 day^-1^ | 0.0038 day^-1^ |
| 15 | 0.0046 day^-1^ | 0.0062 day^-1^ |
| 20 | 0.0022 day^-1^ | 0.0038 day^-1^ |
| 25 | 0.0038 day^-1^ | 0.0030 day^-1^ |
| 30 | 0.0071 day^-1^ | 0.0080 day^-1^ |
| 35 | 0.0207 day^-1^ | 0.0182 day^-1^ |

*2) Additional snail mortality due to infection (α)*

Foster (Foster, 1964) also produced similar data to that of El-Hassan (El Hassan, 1974), showing percentage mortality after 60 days for both infected and uninfected snails. Using equation A1, *δ_S_* and the additional mortality due to infection (*α*) were calculated, producing the following data:

| Temperature  (°C) | Uninfected mortality  (*δ_S_*) | Infected mortality  (*δ_I_*) | Additional mortality  (*α = δ_I_ - δ_S_*) |
| --- | --- | --- | --- |
| 22.85 | 0.0031 day^-1^ | 0.0067 day^-1^ | 0.0036 day^-1^ |
| 24.01 | 0.0033 day^-1^ | 0.0116 day^-1^ | 0.0082 day^-1^ |
| 26.26 | 0.0039 day^-1^ | 0.0260 day^-1^ | 0.0221 day^-1^ |
| 28.07 | 0.0085 day^-1^ | 0.0368 day^-1^ | 0.0283 day^-1^ |

*3) Miracidial mortality rate (δ_M_)*

Anderson *et al* (Anderson et al., 1982) presented data on the mean miracidial life expectancy (in hours) over a range of temperatures. These were converted to daily mortality rates (1/life expectancy) to give the following values:

| Temperature (°C) | Miracidial mortality rate (*δ_M_*) |
| --- | --- |
| 5 | 4.90 day^-1^ |
| 10 | 2.18 day^-1^ |
| 15 | 1.50 day^-1^ |
| 20 | 2.00 day^-1^ |
| 25 | 2.53 day^-1^ |
| 30 | 4.36 day^-1^ |
| 35 | 4.44 day^-1^ |
| 40 | 4.80 day^-1^ |

*4) Adult parasite mortality rate (δ_P_)*

Stirewalt (Stirewalt, 1954) presented data on the proportion of mice containing a varying initial number of worms seven weeks post-infection, which can be used to calculate the mean number of worms per mouse at the end of the seven weeks. In addition, Stirewalt also presented the average number of cercariae that penetrated each mouse at time *t* = 0. Using equation A1, with *t* = 49 days, *N_0_* the initial number of cercariae infecting and *N_t_* the mean number of worms per mouse at the end of the seven weeks gives the following estimates of adult parasite mortality (*δ_P_*):

| Temperature (°C) | Adult schistosome mortality rate (*δ_P_*) |
| --- | --- |
| 23-25 | 0.022 day^-1^ |
| 26-28 | 0.015 day^-1^ |

Estimating developmental rates

*1) Schistosome maturation rate (patency, σ)*

Foster (Foster, 1964) presented data on the time of development to cercarial shedding for *S. mansoni* in *Biomphalaria pfeifferi*. Based on the minimum and maximum periods to shedding presented, the mean maturation rates are:

| Temperature (°C) | Average days to shedding | Maturation rate (*σ*) |
| --- | --- | --- |
| 18 | 57 | 0.0175 day^-1^ |
| 21 | 37 | 0.0270 day^-1^ |
| 22.85 | 34 | 0.0294 day^-1^ |
| 24.01 | 32 | 0.0312 day^-1^ |
| 26.26 | 24.5 | 0.0408 day^-1^ |
| 28.07 | 21.5 | 0.0465 day^-1^ |
| 30.04 | 20 | 0.0500 day^-1^ |
| 31.75 | 17.5 | 0.0571 day^-1^ |

*2) Snail maturation rate (θ_S_)*

El-Hassan (El Hassan, 1974) determined the minimum and maximum incubation periods of snail eggs at between 15°C and 30°C. Based on the average number of days to hatching the mean hatching rate for each temperature is:

| Temperature (**°**C) | Incubation period (days) | Hatching rate (*θ_E_*) |
| --- | --- | --- |
| 15 | 23.5 | 0.042 day^-1^ |
| 20 | 12.5 | 0.080 day^-1^ |
| 25 | 10 | 0.100 day^-1^ |
| 30 | 8.5 | 0.118 day^-1^ |

Estimating birth (production) rates

*1) Snail egg-laying rates (a_S_)*

El-Hassan (El Hassan, 1974) measured the number of eggs laid per snail per day for *B. alexandrina* over 60 days, resulting in daily *per capita* egg production rates of:

| Temperature (**°**C) | Eggs per snail per day (*a*_S_) |
| --- | --- |
| 10 | 0 day^-1^ |
| 12.5 | 0.055 day^-1^ |
| 15 | 0.166 day^-1^ |
| 20 | 0.663 day^-1^ |
| 25 | 0.849 day^-1^ |
| 28 | 0.228 day^-1^ |
| 30 | 0.570 day^-1^ |
| 35 | 0 day^-1^ |

*2) Cercarial production (λ_C_)*

Fried *et al* (Fried et al., 2002) presented data on the number of cercariae released during one hour under various conditions. Based on their standard (incandescent light) conditions, at different temperatures, the daily cercarial production rates (per infected snail) are:

| Temperature (**°**C) | Number of cercariae (*λ_C_*) |
| --- | --- |
| 12 | 504 day^-1^ |
| 25 | 4128 day^-1^ |
| 35 | 8400 day^-1^ |

Estimating infection rates

*1) Miracidial infection of snails (β_S_)*

Stirewalt (Stirewalt, 1954) presented data on the percentage of snails becoming infected following exposure to 1 miracidium in 1 ml water for 24 hours. We can describe the dynamics of this experiment by the equations:

$\frac{dU}{dt}=-MU\beta_{S}$ Eq. A2

$\frac{dI}{dt}=MU\beta_{S}$ Eq. A3

$\frac{dM}{dt}=-MN\beta_{S}$ Eq. A4

where *U* are the number of uninfected snails, *I* the number of infected snails, *N* the total number of snails *(U+I)* and *M* the number of miracidia. Solving these equations provides the following expression for the number of infected snails at time *t*:

$I_{t}=1-e^{-\frac{M_{0}\left[1-e^{-\beta_{S}Nt}\right]}{N}}$, Eq. A5

which can be rearranged to provide an expression for *β_S_*:

$\beta_{S}=-\frac{\ln\left[M_{0}+N\ln(1-I_{t})\right]}{M_{0}Nt}$ Eq. A6

Given *M_0_* (the initial number of miracidia) = 1, *t* = 1 day and *I_t_* is the final number of infected snails at the end of the experiment, this equation provides the following estimates of miracidial infections rates:

| Temperature (**°**C) | Miracidial infection rate (*β_S_*) |
| --- | --- |
| 23-25 | 0.487 day^-1^ ml^-1^ |
| 26-28 | 1.498 day^-1^ ml^-1^ |
| 31-33 | 1.324 day^-1^ ml^-1^ |

*2) Cercarial infection of definitive host (β_H_)*

Stirewalt (Stirewalt, 1954) presented data on the number of worms infecting mice following exposure to 50 cercariae for 1 hour. As before, this experiment can be described by the equation:

$\frac{dC}{dt}=-\beta_{H}CM$ Eq. A7

where *C* is the number of cercariae in the water and *M* the number of mice (which remained constant at 1). This equation can be solved to give the expression:

$\beta_{H}=-\frac{\ln\left(\frac{C_{t}}{C_{0}}\right)}{t}$ Eq. A8

where *C_t_* is the number of cercariae that did not infect and *C_0_* is the initial number of cercariae. Applying this equation to Stirewalt’s data provides the following estimates of miracidial infections rates:

| Temperature (°C) | Cercarial transmission rate (β_H_) |
| --- | --- |
| 23-25 | 52.97 day^-1^ ml^-1^ |
| 26-28 | 71.90 day^-1^ ml^-1^ |
